# How to solve novel problems: the role of associative learning in problem-solving performance in wild great tits *Parus major*

**DOI:** 10.1007/s10071-024-01872-8

**Published:** 2024-04-12

**Authors:** Laure Cauchard, Pierre Bize, Blandine Doligez

**Affiliations:** 1 School of Biological Sciences, University of Aberdeen, Aberdeen, U.K.; 2 Anthropogenic Effects Research Group, Swiss Ornithological Institute, CH-62024 Sempach, Switzerland; 3 Department of Biometry and Evolutionary Biology, CNRS, Univ Lyon, UMR 5558, University of Lyon 1, Villeurbanne, France; 4 Animal Ecology, Department of Ecology and Genetics, Evolutionary Biology Centre, Uppsala University, Uppsala, Sweden

**Keywords:** Accuracy, Cognition, Exploration, Innovation, Learning, Neophobia, Persistence, Personality

## Abstract

Although problem-solving tasks are frequently used to assess innovative ability, the extent to which problem-solving performance reflects variation in cognitive skills has been rarely formally investigated. Using wild breeding great tits facing a new non-food motivated problem-solving task, we investigated the role of associative learning in finding the solution, compared to multiple other non-cognitive factors. We first examined the role of accuracy (the proportion of contacts made with the opening part of a string-pulling task), neophobia, exploration, activity, age, sex, body condition and participation time on the ability to solve the task. To highlight the effect of associative learning, we then compared accuracy between solvers and non-solvers, before and after the first cue to the solution (i.e., the first time they pulled the string opening the door). We finally compared accuracy over consecutive entrances for solvers. Using 884 observations from 788 great tits tested from 2010 to 2015, we showed that, prior to initial successful entrance, solvers were more accurate and more explorative than non-solvers, and that females were more likely to solve the task than males. The accuracy of solvers, but not of non-solvers, increased significantly after they had the opportunity to associate string pulling with the movement of the door, giving them a first cue to the task solution. The accuracy of solvers also increased over successive entrances. Our results demonstrate that variations in problem-solving performance primarily reflect inherent individual differences in associative learning, and are also to a lesser extent shaped by sex and exploratory behaviour.

**Supplementary Information:**

The online version contains supplementary material available at 10.1007/s10071-024-01872-8.

## Introduction

The ability to innovate, i.e., to generate a new behaviour or flexibly adjust an existing behaviour to a new context (Reader and Laland 2003), has been observed in various taxa and proposed as an important trait to explain the evolutionary success of a species (Cantalapiedra et al. 2014; Sol et al. 2016). Indeed, the ability to innovate can allow rapid adjustment to new habitats, such as urban environments (Biondi et al. 2021; Ducatez et al. 2017), for example through the exploitation of new resources (Krützen et al. 2005; Sol et al. 2005). Furthermore, if the benefits of innovating outweigh the costs, innovations can rapidly spread in populations through cultural transmission (Aplin et al. 2015; Ashton et al. 2019; Biro et al. 2003; Krützen et al. 2005), enabling populations to adjust to new conditions before adaptive evolution can take place.

To study the factors affecting the emergence of innovative behaviour and its evolutionary consequences, researchers must devise methods to prompt innovation in order to study it in ‘real time’, as the spontaneous nature of innovation means that it is rarely observed in nature. Therefore, innovation is typically assessed by means of problem-solving tests that have been adapted to the study species’ morphology and physical abilities (Cole et al. 2011; Jacobson et al. 2022; Johnson-Ulrich et al. 2020; Petelle et al. 2023; Rosenberger et al. 2021; Rowell and Rymer 2021). Problem-solving ability can be defined as “the process of overcoming an obstacle via various actions and tools to achieve a goal when the problem solution is not in the species-typical repertoire or socially learned” (Seed and Mayer 2017). Problem-solving tasks are traditionally designed as devices blocking the access to a resource, such as food (but see Cauchard et al. 2017; for an example of non-food motivated task), to investigate how individuals overcome the proposed obstacle using a novel or modified behaviour to solve the problem. However, it remains unclear to what extent problem-solving performance reflects variations in cognitive abilities (and which ones), primarily because of the limited understanding of the cognitive processes that are involved (Amici et al. 2019; Cauchard and Doligez 2023; Cooke et al. 2021; Griffin and Guez 2014; Sol et al. 2012).

Cognitive processes encompass all the mechanisms by which animals perceive, learn and process information from the environment and subsequently act on it (Shettleworth 2010). In humans, problem-solving ability is measured using various psychometric tests that can target more specific cognitive traits than those designed for non-human animals. Human problem-solving ability has been shown to rely on inferential and causal reasoning, exploration, innovation, general intelligence, and several executive functions (i.e., perception, recognition, memory, learning) (Wang and Chiew 2010). However, identifying the mechanisms underlying behavioural responses in non-human animals is much more challenging because a language-based approach cannot be used. Like any behaviour, success or failure to solve a problem is likely to depend on several cognitive processes, whose role and importance might vary with previous experience. To solve a problem, animals need first to perceive and recognize it. Then, they would need to respond appropriately, interacting with the task and processing information. From this moment, memory (Chow et al. 2017; Rowell and Rymer 2021) and learning are involved (Chow et al. 2016), enabling the making of bonds between cues to take decision and to develop the solving strategy. The recent discovery in wild finches of a link between problem-solving and densities of neurotransmitter receptors known to be involved in mammalian cognitive abilities (Audet et al. 2018) supports the hypothesis that problem-solving in non-human animals relies, at least in part, on cognitive processes. Yet, studies investigating problem-solving abilities in non-human animals therefore rarely identified the precise underlying cognitive mechanism(s) (Audet et al. 2018; Chow et al. 2016, 2017; Griffin and Guez 2014; Rowell and Rymer 2021) and rather emphasized the role of other behavioural traits (e.g., reaction to novelty, motivation, persistence), as well as intrinsic (e.g., age, sex, previous experience) and extrinsic (e.g., group size, environmental variation) factors that are mostly determined by non-cognitive processes (e.g., Ashton et al. 2019; Cooke et al. 2021; Jacobson et al. 2022; Petelle et al. 2023; but see Barrett 2014).

To explore how animals solve problems and disentangle the role of both cognitive and non-cognitive factors during problem-solving, we tested wild adult great tits (*Parus major*) on a non-food motivated problem-solving task as they reared their nestlings. The task consisted of a door temporarily attached to the entrance of their nest-box, which prevented them from accessing their nestlings and required them to find a solution (i.e., pull a string) to enter the nest-box to feed their offspring (Cauchard et al. 2013, 2017). We first tested (i) the effects of accuracy (measured as the proportion of contacts with the opening part of the task), as well as behavioural (neophobia, exploration, activity, participation time), intrinsic (age, sex, mass) and extrinsic (year of testing, timing of the season) factors on the ability to solve a new problem-solving task. We then investigated the role of associative learning during problem-solving by (ii) comparing accuracy before and after the first cue to the solution (i.e., first pull of the string that made the door move); (iii) among solvers, investigating changes in accuracy over consecutive entrances. If associative learning contributes to problem-solving efficiency, we expect solvers (i) to show higher accuracy and thus more contacts toward the solving part of the task than non-solvers, and (ii) to persist in doing so especially once potential solving cues arise (Cooke et al. 2021; Overington et al. 2011). Moreover, we expect solvers (iii) to improve their efficiency in solving the task, as reflected by an increase in their accuracy over successive entries.

## Materials and methods

### Study species and study site

The great tit is a passerine bird known for displaying numerous innovative behaviours, such as spontaneous use of new food resources or the ability to solve numerous problem-solving tasks provided in captivity or in the wild (Cauchard et al. 2017; Cole et al. 2011; Estók et al. 2010; Lefebvre 2021). Great tits are secondary cavity nesters that readily breed in nest-boxes from April to June, and both sexes participate in provisioning the nestlings, enabling the use of a non-food motivated problem-solving task attached to the nest-box during nestling provisioning (Cauchard et al. 2013, 2017). Data were collected from a Swedish population of great tits breeding on the island of Gotland (57°10’N, 18°20’E) from 2010 to 2015. Nest-boxes were monitored regularly from the beginning of the breeding season to record breeding success (described in Cauchard et al. 2013). Once nestlings were 10 days old, parents were caught within the nest-box using a swing-door trap or mist nets to be identified or ringed (if previously unringed), sexed and aged (yearling vs. older individuals) according to plumage characteristics (Svensson 1992), and measured (body mass to the nearest 0.1 g, tarsus length to the nearest 0.1 mm).

### Problem-solving test and behavioural measurements

**Fig. 1:**
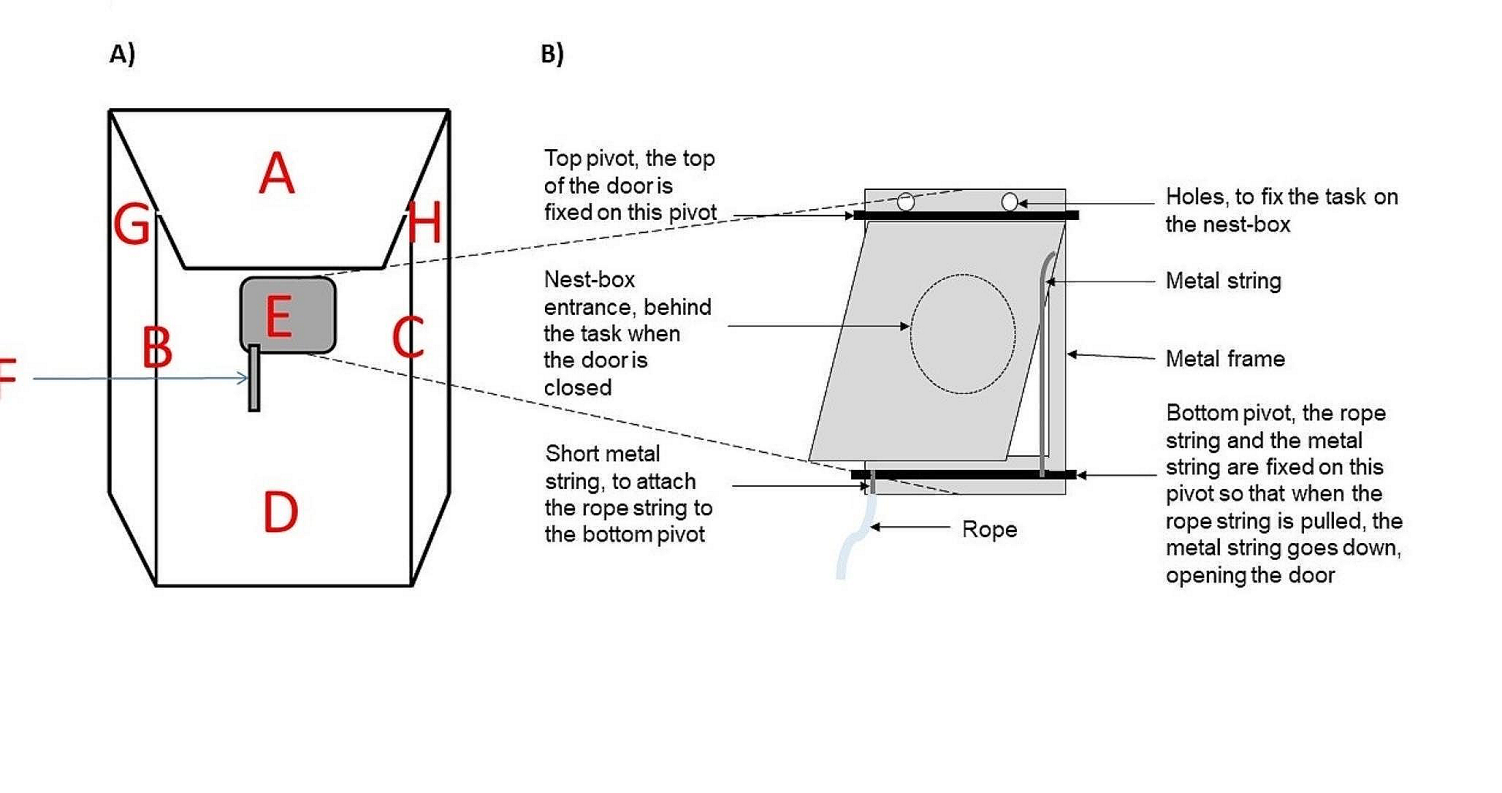
Schematic diagram of the nest-box, its division into 8 contact areas and the non-food motivated problem-solving task. (**A**) Nest-box view of the 8 areas bounded by the wooden parts of the nesting box (A roof, B left corner, C right corner, D front, E task, F string, G left side, H right side; birds are considered as interacting with the task when they have a leg or beak contact with E and F), and (**B**) close-up on the problem-solving task

The problem-solving task consisted of a door placed, on the day of the test, in front of the nest-box entrance hole. The door was closed by default (Fig. 1; described in Cauchard et al. 2013). A bird could only enter the nest-box by using its leg to pull a string hanging under the door that opened it, and by simultaneously sliding its body under the door. The door then closed behind the bird but could be pushed open by the bird from inside the nest-box to exit.

The tests were conducted during the peak of nestling food demand (when nestlings were 6 to 8 days old and between 07:00am – 04:00pm). Immediately before the start of each test, we ensured that nestlings were not too hungry by assessing their begging behaviour (i.e., no intensive begging), and thus that they would potentially endure not being fed for one hour should both parents be non-solvers. If chicks were begging intensively, we cancelled the test and tried again later. Otherwise, we attached the task on the entrance of the nest-box using pins and started the test for one hour, at the end of which the task was removed. We repeated the same procedure on the next day. The test lasted one hour each day over two consecutive days to reduce the length of time chicks would remain unfed per day in the case when both parents were non-solvers while allowing a long enough test duration for birds to be able to explore and learn how to open the door (two hours in total). Thus, in our analyses, problem-solving performance is measured over a two-hour test period split over two consecutive days (corresponding to one observation = statistical unit). Pairs to be tested were selected randomly and spaced at least 200 m apart to avoid social learning. The test was recorded by a camera hidden under a camouflage net, placed approx. 6 to 10 m from the nest-box and facing the entrance of the nest box as much as possible given the vegetation around. No observer was present around the nest box during the test. We confirmed that parents were not disturbed by the camera by checking when analysing the videos that they were not producing alarm calls aimed at the camera. Moreover, on average, birds took 5 min (± 5.5 min) to come back to their nest after we attached the task.

Videos recordings were analysed by several observers who were blind to the hypotheses tested. From the recordings, we scored:

- Problem-solving performance, measured as whether or not the birds succeeded in pulling on the string and entered the nest-box at least up to their shoulders within the two-hour testing period (binary variable: 1 = solver, i.e., succeeded, vs. 0 = non-solver, i.e., failed).
- Neophobia, measured as the time elapsed between the first contact with the nest-box and the first contact with the task within the two-hour testing period (continuous variable, in seconds). We attributed a score of 7200 s, i.e., maximum duration of the two tests performed, to a bird that was observed on the nest-box but never touched the task.
- Exploration, measured as the number of different areas of the nest-box and the task contacted until the first entrance for solvers, or until the end of the test for non-solvers, within the two-hour testing period (discrete variable, from 1 to 8). The nest-box was divided into 6 areas (roof, front, left and right sides, left and right corners) and the task into 2 areas (door and string) (Fig. 1).
- Activity, measured as the total number of areas contacted until the first entrance for solvers, or until the end of the test for non-solvers, within the two-hour testing period (continuous variable).
- Participation time, measured as the time spent on the nest-box during the test until the first entrance for solvers, or until the end of the test for non-solvers, within the two-hour testing period (continuous variable, in seconds).
- Accuracy, measured as the proportion of task-relevant contacts (i.e., contacts with the door and the string) over all contacts with the task and the nest-box either until they enter the nest-box for solvers, or until the end of the test for non-solvers, within the two-hour testing period. Accuracy was thus 0 when a bird never contacted the door or the string, and 1 when a bird only contacted those areas.

### Statistical analyses

We first investigated whether, until the first entrance for solvers or until the end of the test for non-solvers, accuracy, neophobia, activity, exploration, age, sex, body condition (measured as the ratio of mass on tarsus length), participation time, year of testing and timing of the season (explanatory variables) were linked to problem-solving performance (response variable) using generalized linear mixed models (GLMMs). To account for the possibility that the role of accuracy varies between the sexes and with age, we also included in our models the interactions between accuracy and sex or age of individuals. We included as random factors (i) year to account for temporal variation in problem-solving performance between years (e.g., due to yearly environmental conditions), (ii) nest-box identity to account for the lack of independence of pair members and (iii) ring number to account for multiple testing of individuals over years.

Secondly, we examined whether accuracy changed after an individual touched the string leading to the first movement of the door during the first attempt to solve the task. Indeed, if associative learning is involved in solving the problem, we expect solvers to contact more often the solving part of the task following the discovery of a cue leading to the task solution, such as the movement of the door. Hence, we tested whether accuracy (response variable) differed before and after the first door movement (timing group “before vs after”) according to problem-solving performance by including the interaction between timing group and problem-solving performance (explanatory variables), in a linear mixed model (LMM). As in our previous models, year, nest-box identity and ring number were entered as random factors. Because the separation between and after the first door movement may however rely on an arbitrary hypothesis that door movement is a cue used by birds, we also tested the robustness of our results by examining changes in accuracy during the test, comparing results between before and after the middle of the test. This cut-off was placed at mid-test, calculated as half the total number of contacts of the bird with nest-box and test areas until the first entrance for solvers (i.e., half of activity), or until the end of the test for non-solvers (or half + 1 if the number was odd). We thus compared accuracy before mid-test and between mid-test and the first entrance for solvers, and before mid-test and between mid-test and the end of the test, for non-solvers.

Finally, we examined the change in solvers’ accuracy (response variable) over successive entrances (attempt number as explanatory factor) using LMMs. We restricted this analysis to 2015 because accuracy over successive attempts was not extracted from videos for the years before. On average (SE), solvers entered 5.04 ± 0.9 (min-max: 1 to 62 entrances) times their nest-box during the two-hour test period. We thus examined the change in accuracy over the first 5 entrances for birds that entered at least 1 to 5 times. Nest-box identity and ring number were entered as random factors.

All statistical analyses were conducted using R version 4.2.1 (R Development Core Team 2020) and RStudio version 2022.07.2 (R Studio Team 2020). Numerical explanatory variables were scaled to improve the interpretability of model estimates (Schielzeth 2010), and we investigated multicollinearity between explanatory variables using Variance Inflation Factors (VIF) following Zuur et al. (2010). Residuals were visually checked to ensure that model assumptions were met (i.e., homogeneity, normality of residuals). Sample sizes varied between models due to missing data. All tests were two-tailed and *P*-values < 0.05 were considered significant.

## Results

We used data from 884 observations made on 788 different birds (708 birds tested over a single year, 65 birds tested over 2 years, 14 birds tested over 3 years and 1 bird tested over 4 years). The data was collected over 6 years (*N* = 44 birds tested in 2010, 108 birds in 2011, 273 birds in 2012, 116 birds in 2013, 110 birds in 2014 and 231 birds in 2015). In total, the birds solved the problem at least once in 384 of the 884 observations (43% of solving success). Solvers took on average ± SE = 246 ± 17 s to solve the task (min-max: 11 to 4624 s). Furthermore, solvers contacted the task-relevant areas (i.e., string or door) on average 22 times out of 44 total contacts before solving, while non-solvers contacted those areas on average 15 times out of 41 total contacts until the end of the test. Only 25 out of the 500 non-solver birds never touched the task-relevant areas.

### What influences problem-solving performance?

Problem-solving performance was significantly explained by differences in accuracy between individuals: solvers showed a higher accuracy, that is, they contacted the relevant parts of the task (until the first entrance) more frequently than non-solvers (until the end of the test) (mean ± SE = 0.54 ± 0.01 for solvers and 0.35 ± 0.01 for non-solvers; Table 1). Solvers were also more explorative until the first entrance than non-solvers (mean ± SE = 5.13 ± 0.07 areas contacted for solvers and 4.93 ± 0.08 areas contacted for non-solvers; Table 1), and individuals in poor condition (i.e., with a low mass relative to their tarsus length) were more likely to solve the task than individuals in good conditions (mean ± SE = 0.79 ± 0.002 for solvers and 0.80 ± 0.002 for non-solvers; Table 1). Moreover, solvers spent less time on the nest-box until the first entrance than non-solvers until the end of the test (mean ± SE = 246 ± 17 s for solvers and 269 ± 14 s for non-solvers; Table 1). Finally, females were more likely to solve the task than males: 53% of the females (239 out of 451) versus 33% of the males (145 out of 433) solved the task (Table 1), which may be in line with the effect of body condition of problem-solving performance since females had a lower body condition than males during chick rearing (females: mean ± SE = 0.79 ± 0.002, males: 0.80 ± 0.002; *F*_(1,851)_ = 5.27, *P* = 0.022).

Neophobia, activity and age did not affect problem-solving performance (Table 1). Neophobia did not differ significantly between solvers and non-solvers (respectively: mean ± SE = 170.9 ± 34.4 s and mean ± SE = 624.2 ± 69.5 s), nor activity (respectively: mean ± SE = 43.7 ± 2.2 and mean ± SE = 41.2 ± 2.0). Overall, 204 out of 395 yearlings solved the task while 169 out of 465 older great tits solved it. Yearlings also showed a lower body condition than older individuals during chick rearing (yearlings: mean ± SE = 0.79 ± 0.002, old: 0.80 ± 0.002; *F*_(1,850)_ = 19.48, *P* < 0.001), which might have blur the effect of age on problem-solving performance.

**Table 1:** Results of the generalized linear mixed model testing the effects of cognitive (accuracy), behavioural (neophobia, exploration, activity, participation time), intrinsic (age, sex, body condition) and extrinsic (year and day of testing) factors on a non-food motivated problem-solving performance for 728 observations of 647 wild great tits (*Parus major*). The effect of age is expressed as yearling versus older adults (estimate for older adults compared to yearlings here), and the effect of sex as males versus females (estimate for males compared to females here). Significant fixed effects are reported in bold

Problem-Solving Status			
*Predictors*	*Odds Ratios*	*CI*	*p*
(Intercept)	1.27	0.49–3.27	0.627
**Accuracy**	**4.07**	**2.54–6.53**	**< 0.001**
Neophobia	0.88	0.63–1.23	0.467
Activity	1.08	0.81–1.44	0.594
**Exploration**	**1.39**	**1.05–1.82**	**0.019**
Age [yearling]	1.26	0.83–1.90	0.283
**Sex [male]**	**0.51**	**0.35–0.76**	**0.001**
**Body condition**	**0.80**	**0.65–0.98**	**0.030**
**Participation time**	**0.72**	**0.55–0.96**	**0.026**
Day of testing	1.07	0.79–1.44	0.663
Accuracy × Sex [m]	0.72	0.46–1.13	0.157
Accuracy × Age [y]	0.86	0.54–1.38	0.530
*Random Effects*			
σ2	3.29		
τ00 Ring.nb	0.00		
τ00 Nest.ID	0.81		
τ00 Year	1.05		
ICC	0.36		
N Ring.nb	647		
N Nest.ID	439		
N Year	6		
Observations	728		
Margl R^2^ / Cond R^2^	0.272 / 0.535		

### The role of accuracy during problem-solving

**Fig. 2:**
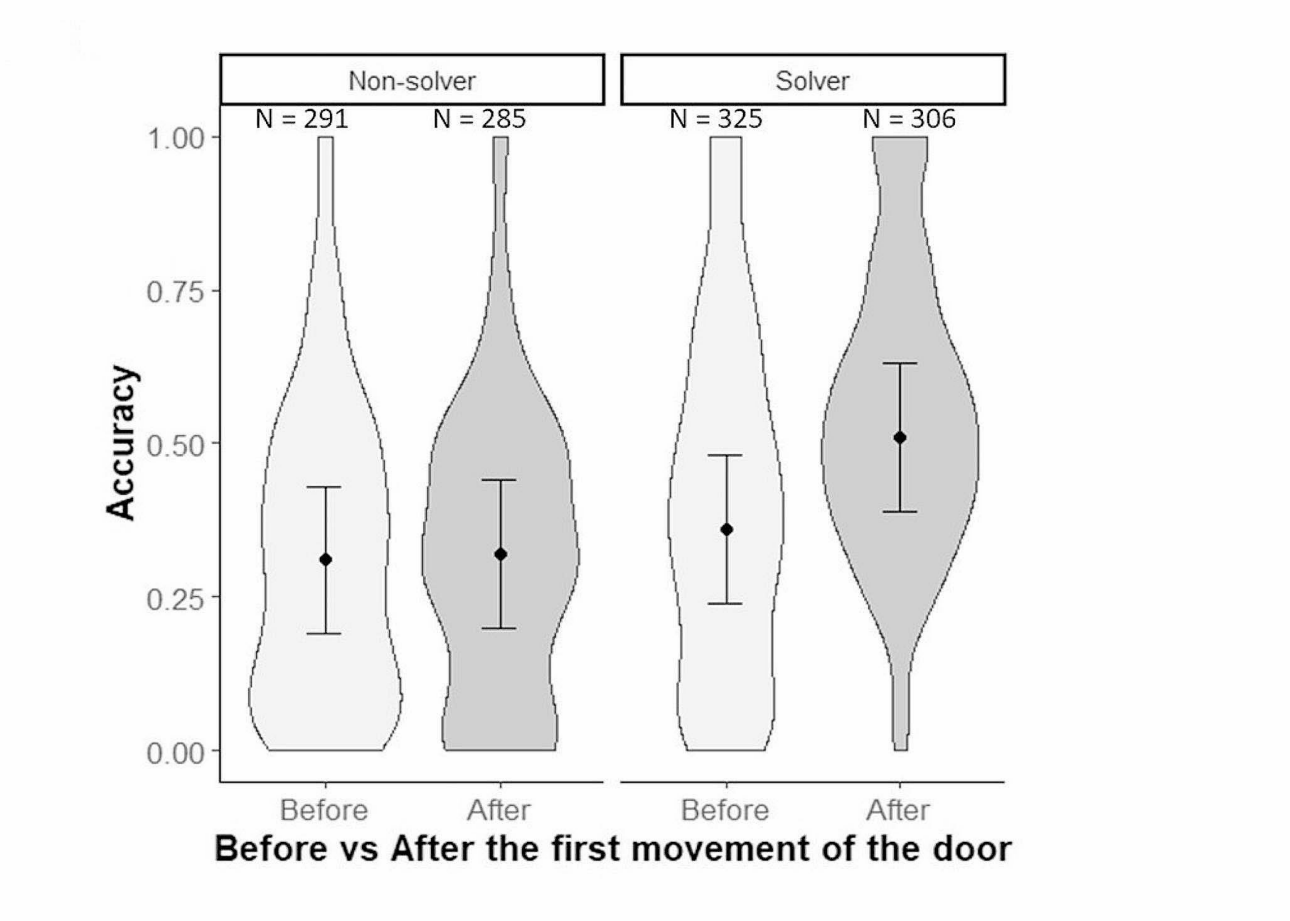
Mean (± SE) accuracy (i.e., proportion of task-relevant contacts over all contacts) compared before and after the first movement of the door until the first entrance for solvers, or until the end of the test for non-solvers, in wild great tits (*Parus major*) faced to a non-food motivated problem-solving task. *N* = 1207 observations from 560 individuals. The distribution of raw data is presented using violin plots while predicted data from the model are presented in black

Accuracy varied before and after the first movement of the door, but only for solvers (interaction between problem-solving performance and timing group [i.e., before vs. after]: *F*_(2,654.6)_ = 5.98, *P* < 0.001; Fig. 2). Accuracy significantly increased after the first door movement in solvers (mean ± SE = 0.36 ± 0.12 before movement; and 0.51 ± 0.12 after movement; Tukey HSD test *P* < 0.001), while it did no change in non-solvers (mean ± SE = 0.31 ± 0.12 before movement; and 0.32 ± 0.12 after; Tukey HSD test *P* = 0.988; Fig. 2). Accordingly, solvers and non-solvers exhibited significant differences in accuracy after the first movement of the door (Tukey HSD test *P* < 0.001) (Fig. 2).

Removing from our analyses the 25 birds that never touched the task-relevant parts did not change qualitatively our results. The results also remained qualitatively unchanged when we compared accuracy before and after mid-test (see Supplementary Information).

### The role of accuracy during successive attempts

**Fig. 3:**
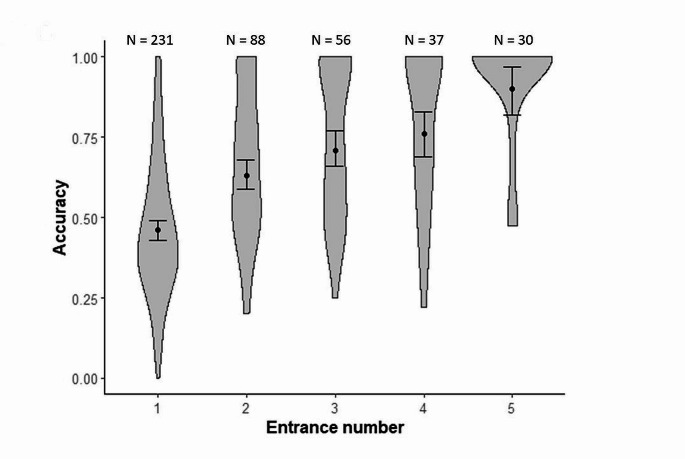
Mean (± SE) accuracy (i.e., proportion of task-relevant contacts over all contacts) prior to successive entrances for solvers, in wild great tits (*Parus major*) faced to a non-food motivated problem-solving task. *N* = 442 observations from 231 individuals that entered at least once in 2015 (data not available for the other years). The distribution of raw data is presented using violin plots while predicted data from the model are presented in black

In solvers, accuracy increased significantly over the first five entrances (*F*_(4,283.9)_ = 47.62, *P* < 0.001; Fig. 3). Post hoc analyses showed that accuracy increased significantly from entrance 1 (mean ± SE = 0.46 ± 0.01) to entrance 2 (mean ± SE = 0.63 ± 0.02; Tukey HSD test *P* < 0.001). Despite a slow further increase, accuracy did not significantly differ from entrance 2 to entrance 3 (mean ± SE = 0.71 ± 0.03; Tukey HSD test *P* = 0.12; Fig. 3), and from entrance 3 to entrance 4 (mean ± SE = 0.76 ± 0.03; Tukey HSD test *P* = 0.78). Then accuracy was again significantly higher from entrance 4 to entrance 5 (mean ± SE = 0.90 ± 0.04; Tukey HSD test *P* = 0.021; Fig. 3).

## Discussion

While animals’ cognitive abilities depend on one or more brain structures and associated cognitive processes, it is also widely acknowledged that cognitive performance can be impacted by various other factors (e.g. Buchanan et al. 2013; Reichert et al. 2020; Roth et al. 2012; Taylor et al. 2012). Problem-solving is no exception, with multiple studies showing that problem-solving performance can be related to sex (e.g. Laland and Reader 1999), age and experience (e.g. Hopper et al. 2014), response to novelty (e.g. Biondi et al. 2010) or persistence (e.g. Daniels et al. 2019), among others (review in Griffin and Guez 2014). In this study, we used a large number of observations of wild great tits solving a new problem to show that inter-individual differences in problem-solving performance are explained by inherent individual differences in associative learning measured as a change in accuracy after individuals had the opportunity to acquire information about the task solution. Problem-solving performance also depended on other factors, here sex and exploratory behaviour.

### Effects of associative learning on problem-solving performance

To test whether the resolution of our problem-solving task had a cognitive basis, we first compared how solvers and non-solvers interacted with the task, and then we examined how these interactions changed after solvers and non-solvers received a first cue to the solution (i.e., first contact with the string that opened the door). Our results showed not only that (i) solvers generally concentrate their attention on the solving part of the task more than non-solvers, but also that (ii) solvers persisted in shifting their attention even more towards the opening door following a cue leading to the solution, making less and less non-opening contacts, whereas non-solvers did not change their initial strategy and percentage of non-opening contacts. Indeed, solvers markedly increased their contacts towards the task-relevant parts following the appearance of a cue leading to the solution, reaching 51% of ‘correct’ contacts, whereas non-solvers did not change their strategy and stayed around 32% of ‘correct’ contacts. Finally, when looking at repeated entrances, solvers also increased their contacts towards the task-relevant parts between further entrances. Our findings therefore suggest that problem-solving performance is partly driven by associative learning, highlighting a strong cognitive basis for this trait. Indeed, the ability to make connections between the cue and the solution was distinct for solvers and non-solvers: solvers increased their goal-oriented behaviour towards the string after the first opening of the door, thus reinforcing the behaviour leading to the solution, while non-solvers did not seem to associate the string with the movement of the door. Alternatively, solvers and non-solvers might not (or not only) differ in their ability to make associations, but (also) in their ability to pay attention: giving attention to the movement of the door may be essential before learning the association between the movement and the reward. Since the reward in our case, i.e., access to the chicks, might have been less directly beneficial for the participant than a food reward used in traditional cognitive tests, it might have increased the difficulty of paying attention and establishing links between the cue and the solution.

Finally, although other, non-cognitive, traits explained inter-individual differences in problem-solving performance (discussed below), the odd-ratios obtained clearly indicate that associative learning is by far the most important factor to find the solution (see Table 1: the odd-ratio for accuracy is > 3 times higher than other significant factors). Our results support the hypothesis that even if problem-solving performance can be affected by non-cognitive factors, cognitive processes such as associative learning or memory are essential to solve a problem (Cooke et al. 2021; Overington et al. 2011; Rowell and Rymer 2021). A recent study comparing 23 bird species (Audet et al. 2023) showed that performance on several problem-solving tasks was strongly associated with vocal learning complexity and brain size, further supporting the importance of various cognitive processes in problem-solving performance. Taken together, these results thus confirm the role of cognitive traits in problem-solving performance and downplay the recent focus on non-cognitive traits as major factors on problem-solving performance (Rowe and Healy 2014).

### Effects of non-cognitive traits on problem-solving performance

In our study, the most important non-cognitive trait explaining inter-individual differences in problem-solving performance was exploratory behaviour, followed in order of importance by body condition, participation time and sex. Exploration was categorized here as a non-cognitive trait, even though it may also be related to cognitive processes (Carere and Locurto 2011; Guillette et al. 2010; Range et al. 2006), like most behavioural responses. Yet, these cognitive processes are not identified as such, and most likely interact with other (e.g., physiological) processes to shape the integrative resulting behavioural response measured as exploration. Our results show that solvers were more explorative, even if they spent less time on the nest-box, than non-solvers, whereas neophobia did not influence problem-solving performance. A widely accepted assumption posits that birds need to explore all areas of a problem to find out which interactions have a relevant consequence, and that neophobia - the fear of novelty - should thus lower problem-solving performance (Greenberg 2003). However, the literature presents contradictory findings on those links. Some studies support the hypothesis that exploratory behaviour predicts problem-solving success (Benson-Amram and Holekamp 2012; Griffin and Guez 2014; Wat et al. 2020) while others do not (Biondi et al. 2010; Cole et al. 2011; Grunst et al. 2020). The same applies to neophobia (Benson-Amram and Holekamp 2012; Biondi et al. 2010; Cole et al. 2011; Griffin and Guez 2014). This lack of consistency is likely explained by the fact that studies varied not only in the study species used, for which responses to novelty can have different impact on behaviour depending on various factors such as environmental pressure, but also in their methodology to measure behavioural traits (i.e., tests and variables used, sample size etc.). Our results are nonetheless partly in line with those from Cole et al. (2011) who tested 570 wild great tits in captivity and found no effect of neophobia and exploration on a food motivated problem-solving performance. Taken together, these results and ours, both performed using high numbers of great tits tested on two different problem-solving tasks, suggest that neophobia does not seem to influence innovation in this species, but exploration may play a role at least in the wild.

Body mass, and in turn, condition, might also influence problem-solving performance if the task’s solution relies on physical abilities, or if the motivation used to encourage solving a problem is directly linked to the internal state, like in food-motivated tasks (van Horik and Madden 2016). In our study, individuals with a lower mass relative to their tarsus length were more likely to solve the task than heavier individuals. Taking into account that solving our task requires pulling a string, one could have expected heavier individuals to be more likely to solve the task if physical abilities were blurring problem-solving performance. This was however not the case is our study. Importantly, the relative mass measures in this species is far from clear: although they are usually used as a condition index as we did here (Labocha and Hayes 2012), being heavier - indicating more fat reserves - for an adult non-migratory bird is not necessarily beneficial. More reserves might indeed mean more effort during flight to carry this extra mass (Witter and Cuthill 1993) and increase predation risk (Brodin 2000). In adult great tits, body mass showed no clear relation with fitness measures such as survival (Kilgas et al. 2006). Moreover, the costs and benefits of carrying fat reserves might change according to the ability to predict food resources availability: fat reserves might be needed for birds that cannot make sure that they will have sufficient food intake, but not for birds capable of guarantying their intake (Cornelius et al. 2017). In great tits, it has been found that higher problem-solving performance was linked to higher food provisioning to nestlings (Cauchard et al. 2017). Problem solver might thus choose to remain lighter to decrease the costs of high fat reserves. Futures studies should investigate the link between cognitive abilities and fat reserves in relation to the ability of individuals to predict food availability in their environment, e.g., by assessing foraging strategies.

Predictions about sex-dependent effects on problem-solving performance tend to vary across species depending, most of the time, on which sex is more motivated to access resources. For example, in guppies, females were more likely to innovate than males, reflecting parental investment asymmetries in this species and a greater motivational state in females (Laland and Reader 1999). However, no effect of sex on problem-solving performance has been reported in wild spotted hyenas (*Crocuta crocuta*) where females are dominant (Benson-Amram and Holekamp 2012). This is why many studies restrict their test to a single sex, which reduces the sample size but avoids any risk of misinterpretation (e.g. Overington et al. 2011; Rochais et al. 2021). In our study, female great tits were more likely to solve the task than males. No difference between sexes in problem-solving was however reported in another study on great tits using another task motivated by food and captive conditions (Cole et al. 2011). Our task may have been intrinsically more motivating for females than males because the reward (i.e., access to nestlings) is directly related to parental care. Even if great tits have biparental care during nestling provisioning, females can be considered to invest more in their current offspring than males because they lay and incubate eggs and brood hatchlings alone, and because males may modulate their provisioning behaviour depending on their certainty of paternity.

Motivation, defined as the process that initiates, guides, and maintains goal-oriented behaviours (Helms 2000), is often reported as a factor blurring cognitive performance (Cooke et al. 2021; Laland and Reader 1999; Sol et al. 2012). For example, the ‘necessity drives innovation’ hypothesis states that innovations should occur when individuals are in need (Reader and Laland 2003). Thus, in the case of food innovations, access to food should drive innovation ability and young/subordinate individuals with less access to food (Kawai 1965) or individuals with higher internal needs (Laland and Reader 1999) should show a higher probability to innovate. Traditionally, food deprivation is used to ensure participation and is assumed to standardize participants’ motivation and thus control for its confounding effect on cognitive performance (Cooke et al. 2021; van Horik and Madden 2016). We used in this study the access to nestlings as a motivation and experimentally showed in a previous study that brood size did not influence problem-solving performance (Cauchard et al. 2017). Because birds were here free to participate or not, we used participation time (i.e., the time spent on the nest-box until the first entrance for solvers, or until the end of the test for non-solvers) as another measure of motivation and showed that it was not a factor determining solving success. Solvers actually spent less time on the nest-box during the test than non-solvers, even if they showed a higher exploration score (but no difference in activity, i.e., the total number of areas contacted, thus movements) compared to non-solvers. These results exclude the possibility that solvers were successful because they had more time, as birds were free to interact with the task or not. Together with our previous study (Cauchard et al. 2017), these results suggest that inter-individual differences in motivation within a sex category are not driving problem-solving performance.

The other intrinsic (age) or extrinsic (year, date) variables tested in this study did not influence problem-solving performance. Age has been regularly hypothesized to influence problem-solving performance, either because young differ from adults in their free time/energy to investigate a new problem (e.g., Biro et al. 2003) or in their ability to do so (e.g., Sonnenberg et al. 2019). Our results did not show any link between age and problem-solving performance, although our data also indicate that yearlings showed a lower body condition than older individuals during chick rearing, which might have blurred the effect of age on problem-solving performance. Moreover, we would like to be cautious here because we have used the age category assigned in the field on the basis of plumage characteristics (Svensson 1992) in our analyses. This measure might be both prone to errors, and only separates two main categories (i.e., Yearling versus Old, compared to using a continuous variable such as chronological age), which may blur a potential effect of age on problem-solving performance. Future studies investigating this effect of age would require using chronological age to more efficiently test for its link with cognitive ability. Finally, even though meteorological conditions varied both between years and within seasons, these variables did not influence problem-solving performance in our study. More refined meteorological measures, such as mean temperature/rainfall, or environmental quality measures, such as caterpillar resource availability might also help to investigate more closely whether environmental conditions may affect problem-solving ability.

In conclusion, our study, based on a comprehensive list of cognitive and non-cognitive traits and supported by a substantial sample size, shows that accuracy has a major influence on problem-solving performance in our population of great tits, confirming the main role for cognitive processes like perception, attention and associative learning during problem-solving.

## Electronic supplementary material

Below is the link to the electronic supplementary material.

Supplementary Material 1

## Data Availability

The dataset for this study will be available in Zenodo upon publication [LINK].
