# Supplementary material for: How to solve novel problems: the role of associative learning in problem-solving performance in wild great tits Parus major
Source: Anim Cogn. 2024 Apr 12;27(1):32. doi: 10.1007/s10071-024-01872-8 (PMC11014811; doi:10.1007/s10071-024-01872-8)
Supplement: Supplementary file 1 — Supplementary Material 1 [file 10071_2024_1872_MOESM1_ESM.docx]

**Supplementary Information for**

How to solve novel problems: the role of associative learning in problem-solving performance in wild great tits *Parus major*

Laure Cauchard^1,2*^, Pierre Bize^1,2,a^, Blandine Doligez^3,4,a^

^1^ School of Biological Sciences, University of Aberdeen, Aberdeen, U.K.

^2^ Anthropogenic Effects Research Group, Swiss Ornithological Institute, CH-62024 Sempach

^3^ CNRS, Univ Lyon, UMR 5558, Department of Biometry and Evolutionary Biology, University of Lyon 1, Villeurbanne, France

^4^ Animal Ecology, Department of Ecology and Genetics, Evolutionary Biology Centre, Uppsala University, Uppsala, Sweden

^a^ These authors share senior authorship

*** Correspondence:** [laure.cauchard@gmail.com](mailto:laure.cauchard@umontreal.ca)

**The role of accuracy during problem-solving: changes before and after mid test**

To test for robustness of our results, we examined changes in accuracy before and after mid test between solvers and non-solvers (beside changes before and after the first cue; see Figure 1 and results in the article). Mid test was calculated as half of the total number of areas contacted (or half + 1 if the number is odd) until the first entrance for solvers or until the end of the test for non-solvers.

Accuracy varied before and after mid test, and again only for solvers (interaction between problem-solving performance and mid group: *F*_(2, 563.4)_ = 4.89, *P* < 0.001; Fig. S1). Thus, solvers’ accuracy increased significantly after mid test (mean ± SE = 0.47 ± 0.06 before mid test; and 0.59 ± 0.06 after mid test; Tukey HSD test *P* < 0.001), while non-solvers’ accuracy did not differ before and after mid test (mean ± SE = 0.32 ± 0.06 before mid test; and 0.36 ± 0.05 after mid test; Tukey HSD test *P* = 0.21; Fig. S1).


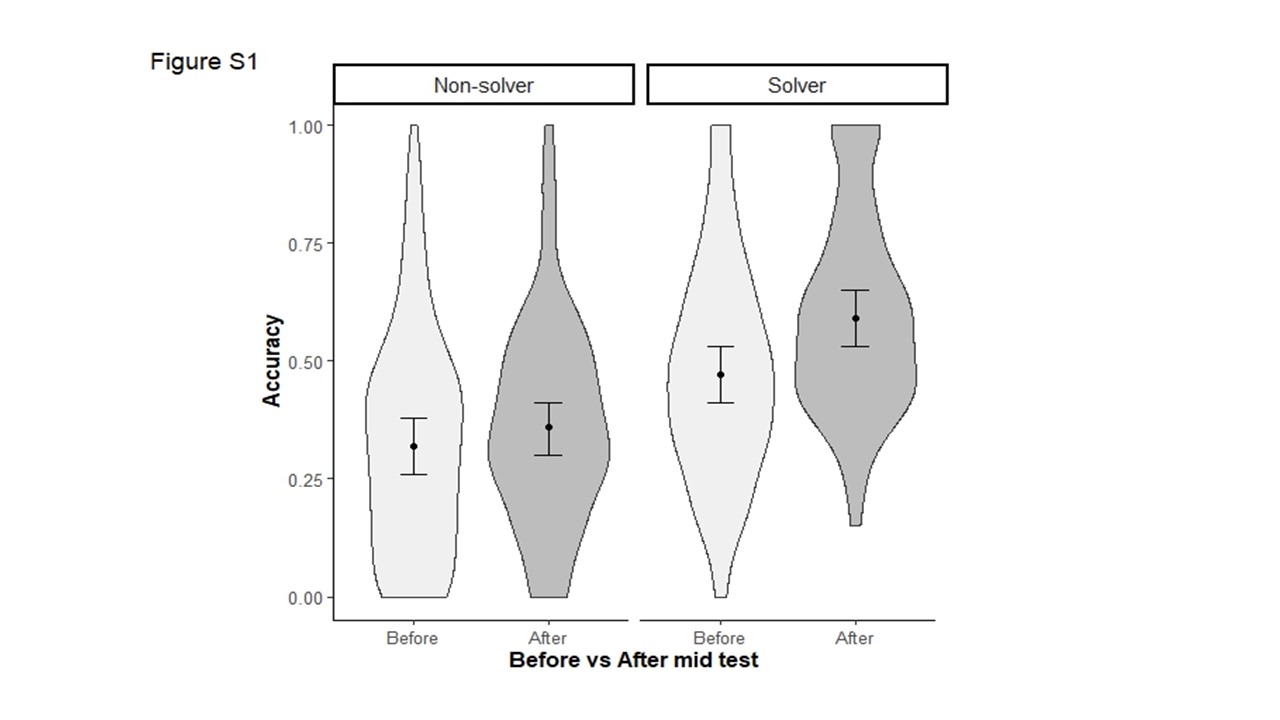


**Fig. S1: Mean (± SE) accuracy before and after mid test for solving and non-solving wild great tits (*Parus major*) faced with a non-food motivated problem-solving task.** Data are from 537 individuals. The distribution of raw data is presented in violin plots, while predicted data from the model are presented in black.
